# Dermatological Manifestations of COVID-19 in Patients Reporting to a Tertiary Care Hospital in Rawalpindi, Pakistan

**DOI:** 10.7759/cureus.18973

**Published:** 2021-10-22

**Authors:** Anjum Muhammad, Nadia Iftikhar, Asher Mashhood, Gurnam Virdi, Hafeez Ud Din, Afnan Akbar, Bilal Ahmad, Abrar Khalid

**Affiliations:** 1 Dermatology, Pak-Emirates Military Hospital, Rawalpindi, PAK; 2 Dermatology, Academy of Aesthetic Medicine, Sheffield, GBR; 3 Histopathology, Armed Forces Institute of Pathology, Rawalpindi, PAK; 4 Basic Sciences, Army Medical College, Rawalpindi, PAK; 5 Endocrinology and Diabetes, Pak-Emirates Military Hospital, Rawalpindi, PAK

**Keywords:** covid 19, dermatological manifestaion, limb ischemia, covid and skin, histological features

## Abstract

Introduction

The clinicopathological description of dermatological manifestations of COVID-19 leaves much to be desired. There is a need to determine their association with disease severity, outcome, and other clinical variables.

Objectives

The objectives of this study are to record and histopathologically examine the cutaneous manifestations of COVID-19 and correlate these to age, disease severity, and mortality.

Methods

All confirmed COVID-19 patients admitted to a single tertiary healthcare hospital in Rawalpindi, Pakistan, were included. Their diseases were classified as mild, moderate, severe, and critical. The recent onset skin eruptions in these patients were recorded via photographs along with relevant clinical data. The photographs were independently reviewed by a group of three dermatologists without knowledge of the clinical information. The skin manifestations were divided into disease-specific and nonspecific categories using an already defined algorithm. Histopathological examination of skin manifestations was conducted.

Results

A total of 23% (n=47) had “new” skin manifestations. Specific skin findings were seen in 21.6% (n=44), which consisted of ecchymosis/purpura in 50% (n=22), maculopapular exanthem in 18% (n=8), livedo reticularis in 16.2% (n=7), ischemia/gangrene in 16.2% (n=7), perniosis in 15.9 % (n=7), vesiculo-bullous rash in 9% (n=4) and urticaria in 4% (n=1). Non-specific findings were seen in 6% (n=13) and included bedsores, dermatitis passivata, dryness, herpes labialis, oral ulcerations, and nasogastric tube-induced ulcerations. There was a significant association (p=0.03) between disease severity and specific skin lesions. Ischemia/gangrene was significantly associated with COVID-19 disease severity and mortality. Vesiculobullous lesions were associated with higher mortality, though not with disease severity. Livedo reticularis had a higher-than-expected count in critical disease, albeit statistically insignificant. The association of maculopapular exanthem and ecchymosis/purpura with severe/critical disease was statistically insignificant. Urticaria was significantly associated with low disease severity. Mean age with specific manifestations was 56.86 ± 15.81 and with nonspecific/without any manifestations was 42.58 ± 16.96, a highly significant difference, with p-value < 0.001. Old age (>60 years) was significantly associated with ecchymosis (p=0.038), maculopapular exanthem (p=0.021), and vesiculo-bullous rash (p=0.029). Histopathology varied according to the type of skin lesion.

Conclusions

Dermatological manifestations coexist in many patients and tend to appear more in severe cases of COVID-19 among the older age group and only minimally in mild/moderate cases. Their presence could help set prognostic criteria of COVID-19 disease in the future.

## Introduction

COVID-19 was first detected in the Chinese city of Wuhan in December 2019 as pneumonia that involved multiple organ systems [1]. The newfound virus would promptly spread across swaths of the planet and be declared a global pandemic shortly after.

As part of the multisystem involvement that COVID-19 encompasses [2], the skin can exhibit various manifestations. Descriptions of the cutaneous manifestations of COVID-19 have been attempted by various studies across the globe showing fluctuating frequency, types, extent, and association to the disease severity. The frequency of dermatological manifestations may vary from 0.2% to 20.4% [2-5]. These include maculopapular exanthem, urticarial, vesicular/bullous, perniosis ecchymosis/purpura, livedo reticularis, acral ischemia/gangrene, and maculopapular rash with enanthem. COVID-19 may also cause non-specific dermatological manifestations that result from various treatment sequelae, prolonged in-hospital care, and poor skin hygiene of patients [6,7] while being isolated for extended durations.

The limitations of these studies were variable; some were individual case studies, while others had small sample sizes [8,9] or lacked adequate pictorial data [10] due to safety concerns related to the highly contagious virus [3]. Even fewer studies performed the histological examination of the lesions.

Our study attempts to corroborate the previously detailed classifications and descriptions of the cutaneous manifestations of COVID-19, describes their histological features, and attempts to relate them with age, disease severity, and mortality.

## Materials and methods

All confirmed COVID-19 patients (with laboratory confirmation of SARS-CoV-2, irrespective of the severity of their clinical signs and symptoms) admitted in a tertiary inpatient health care facility in Rawalpindi, Pakistan were included. All patients who showed recent-onset skin eruptions were included. Their diseases were classified as mild, moderate, severe, and critical as per WHO guidelines [11].

This descriptive study was conducted in July 2020 for a duration of one month. A specially designed form was used for data collection and pictures were taken of all the skin manifestations using full personal protective equipment protocol.

The photographs were independently reviewed by a group of three dermatologists without knowledge of the clinical information. The skin lesions were then classified using an algorithm for the classification of cutaneous manifestations of COVID-19 [12]. Analysis of descriptive data and distribution tests was done using SPSS Statistics 23 (IBM Corp., Armonk, NY).

The study was approved by an ethical committee (A/28/180/EC/2020). All patients, or their next of kin in case of minors/critically ill, gave their informed consent to participate and explicit consent to use their pictures in publications and perform biopsies when needed.

Complete blood picture including eosinophilic counts was performed to rule out the possible association of the skin manifestations with the drugs administered during the treatment. Histopathological analysis was carried out on biopsies.

## Results

We observed 204 cases during the peak of the epidemic in Pakistan. The patients' ages ranged from 13 years to 93 years. The median age was 45.5 years. Patients above 60 years of age constituted 27% (n=55) of the sample. The rest 73% (n=149) were below 60 years. Males accounted for 88.2% (n=180), while female, 11.8% (n=24) of cases. Case fatality was 4.4% (n=9). Around 92% (n=188) were discharged on recovery and 3.4% (n=7) patients remained under treatment till the compilation of this data. Also, of the COVID-19 patients, 60% (n=114) had mild, 18% (n=38) had moderate, and 17.6% (n=36) had severe, while 7.8% (n=16) was critical according to their clinical and laboratory findings. No deaths were reported in mild and moderate disease, while 2.6% (n=1) patients died in severe and 50% (n=8) died in the critical disease category. Around 29% (n=60) of our patients were comorbid, the most frequent being diabetes (15.4%, n=27), hypertension (18.3%, n=37), ischemic heart disease (11.2%, n=23) and COPD/asthma (4.5%, n=9). Combinations of two or more comorbidities were seen in 13.2% (n=27).

**Figure 1:**
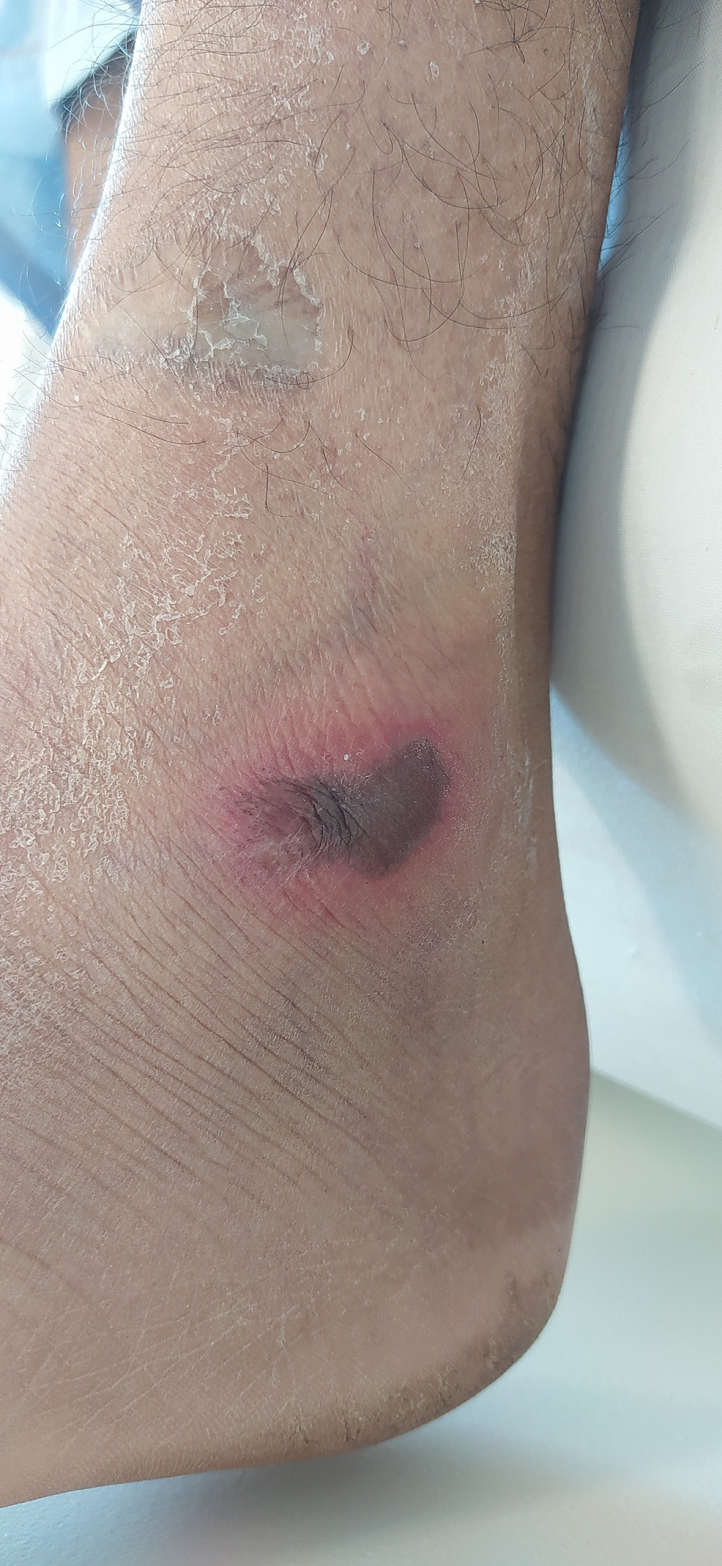
Bullous rash on the lateral malleolus of a critically ill young male.

**Figure 2:**
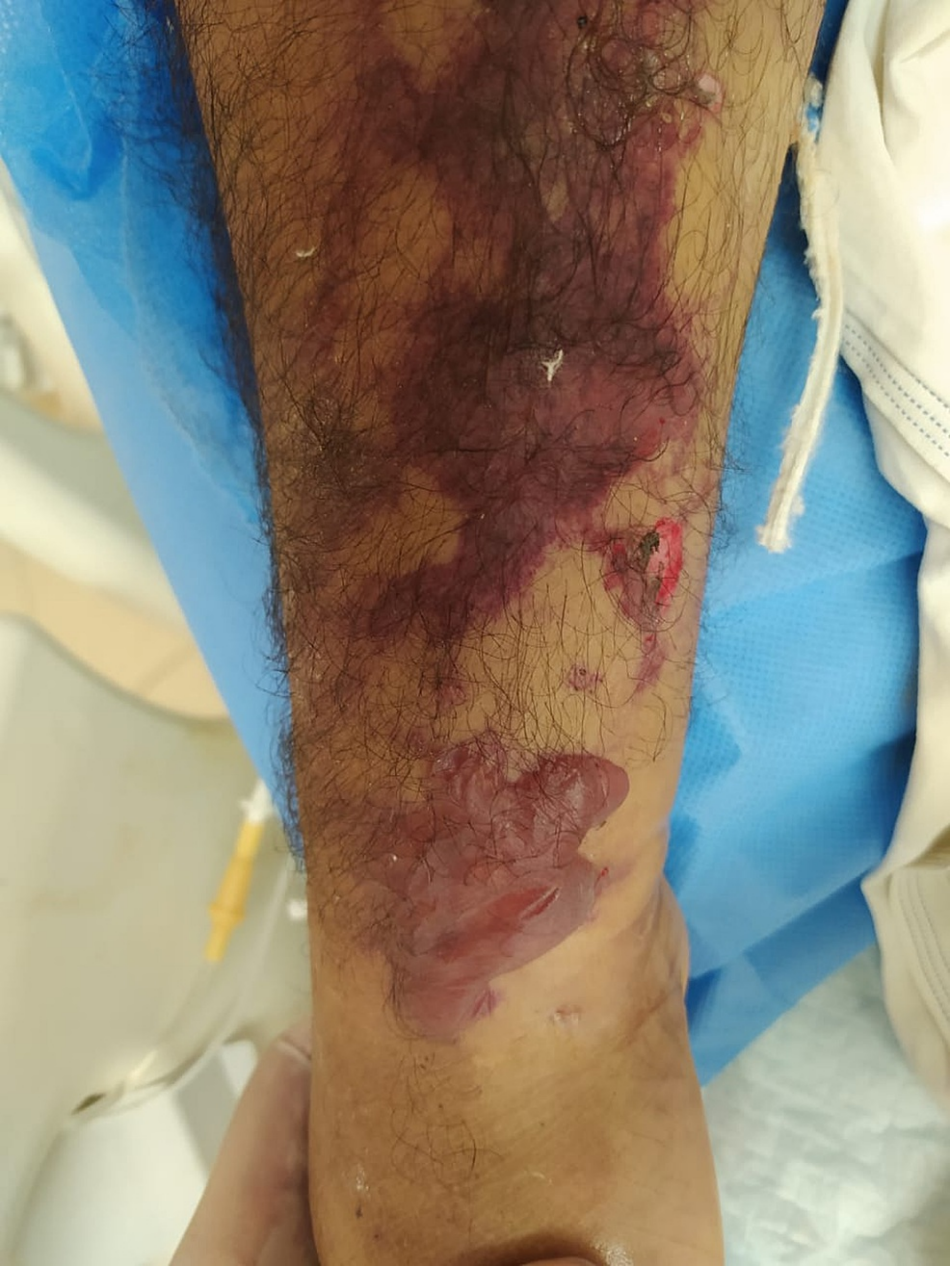
Vesiculobullous rash with ischemia on the lower limb of a critically ill middle-aged male.

**Figure 3:**
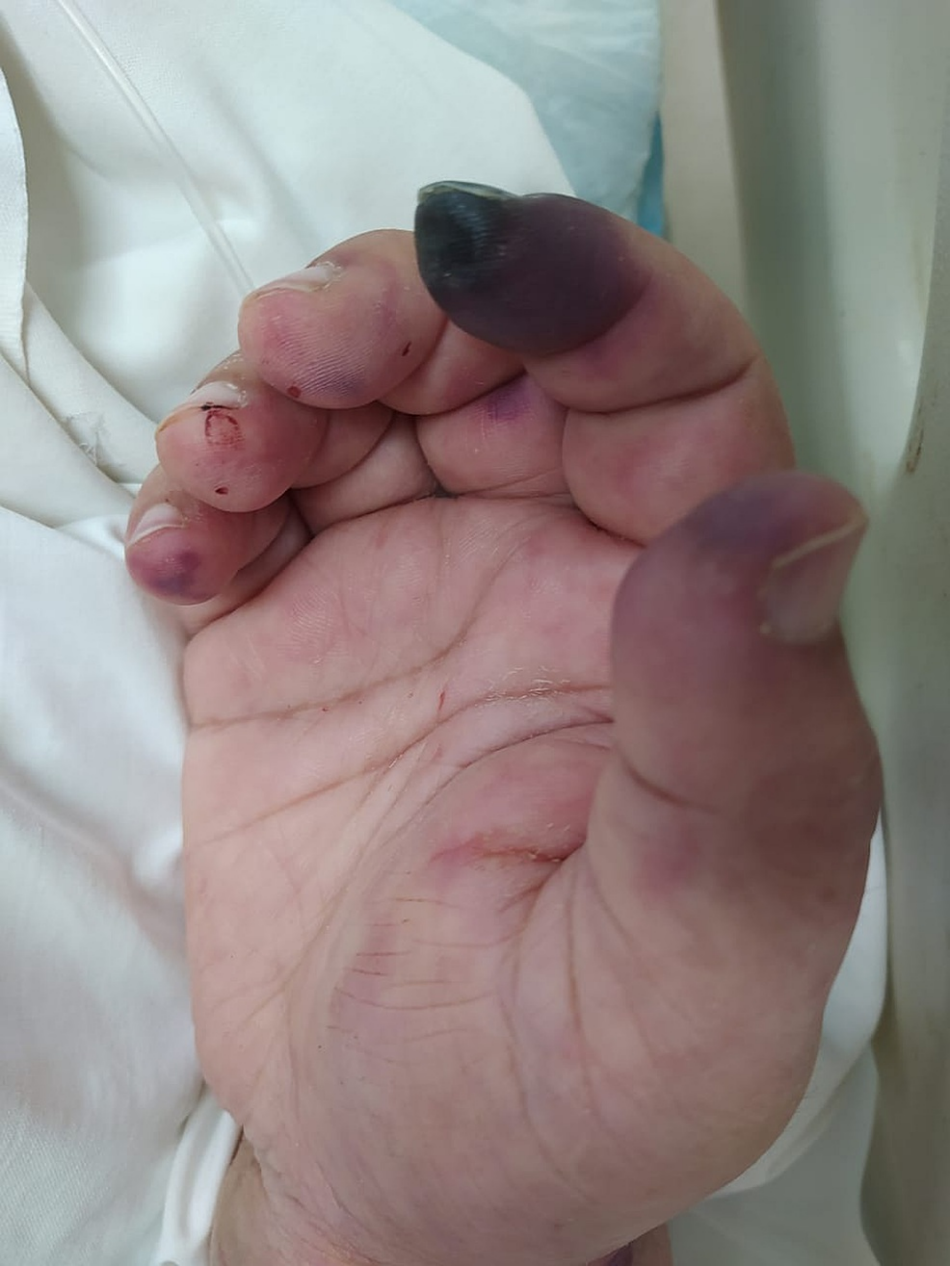
Digital ischemia and perniosis in a critically ill middle-aged female.

**Figure 4:**
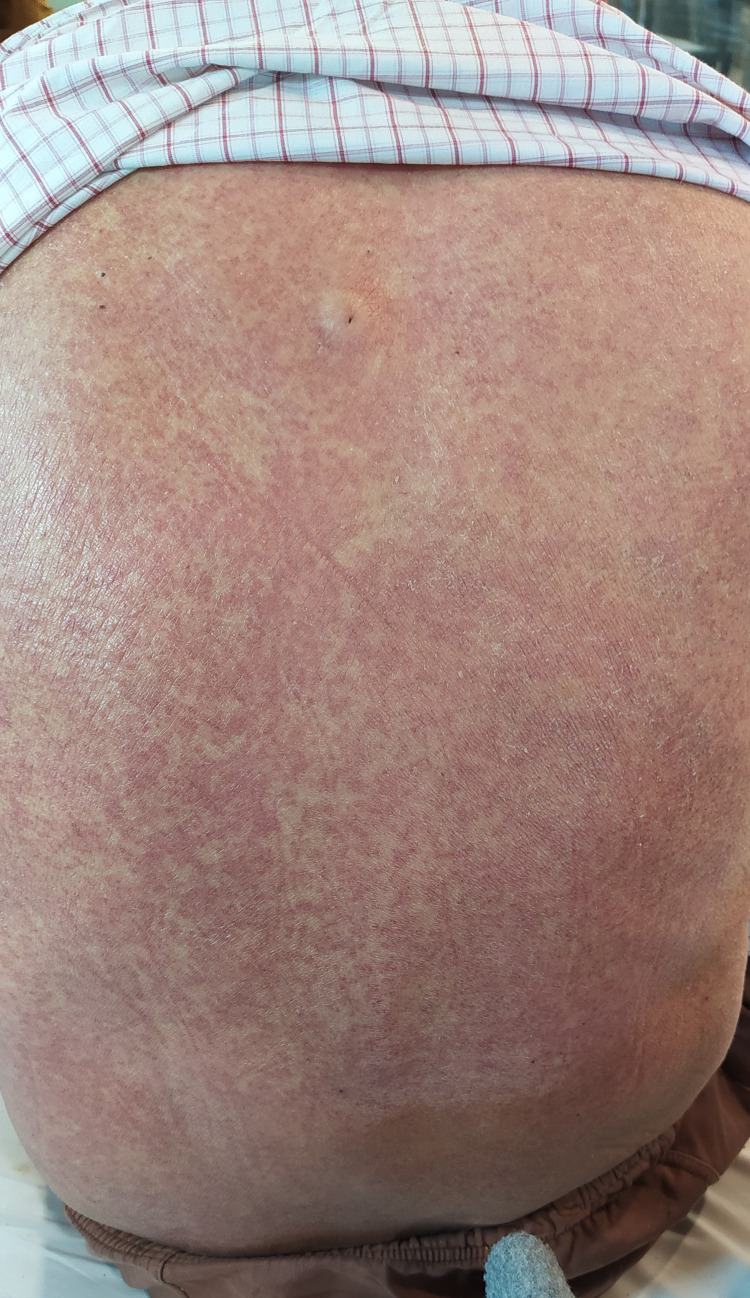
Blanchable maculopapular rash on the back of an elderly male.

Image review and data analysis, lead to the description of two major clinical classes i.e. specific and nonspecific skin findings. The specific findings included seven major clinical patterns namely vesiculobullous, digital ischemia/gangrene, maculopapular exanthem, and livedo reticularis (Figures 1-4). Additional findings included ecchymosis/purpura, perniosis, and urticarial manifestations. Non-specific skin manifestations included bedsores, dermatitis passivata, dryness, herpes labialis, oral ulcerations, and nasogastric tube-induced ulcerations.

Around 23% (n=47) had some or other “new” skin manifestations during the course of their infection: these were skin manifestations that appeared on the patients' skin within the incubation period following contact with an infected person or following the development of other signs and symptoms of COVID-19. The onset of skin lesions ranged from five days prior to COVID-19 diagnosis to 26 days after diagnosis. Only one patient (2.2%) reported palmar livedo reticularis five days prior to diagnosis. Lesions appeared in the first, second and third week of illness in 27.2% (n=12), 36.3% (n=16), and 34% (n=15) of the patients respectively. Specific skin findings were seen in 21.6% (n=44). Amongst these, 16.7% (n=34) patients had specific manifestations only, while 1.5% (n=3) had nonspecific skin manifestations only. A combination of both was seen in 4.9% (n=10). No skin findings were seen in 77% (n=157) of patients. The summary of specific dermatological manifestations and their histopathology is shown here (Table 1).

**Table 1:** Descriptive characteristics of the patients with specific skin manifestations along with biopsy findings. DM: diabetes mellitus; HTN: hypertension; IHD: ischemic heart disease; SJS/TEN: Stevens-Johnson syndrome/toxic epidermal necrolysis; COPD: chronic obstructive pulmonary disease; HIV: human immunodeficiency virus, HCV/DCLD: Hepatitis C virus/decompensated liver disease

Age	Sex	Severity of Disease	Comorbidities	Onset of Rash	Type of Skin Rash	Biopsy Findings	Disease Outcome
92	M	Critical	HTN	09	Ecchymosis/purpura	-	Discharged
40	M	Severe	Nil	08	Perniosis, ecchymosis	-	Discharged
67	F	Severe	DM, HTN	07	Maculopapular/exanthum	Orhtokeratosis, thin necrotic epithelium stripped from dermis, perivascular lymphocytic infiltration in dermis with mucinous degeneration.	Under treatment
60	M	Critical	Nil	09	Perniosis + livedo reticularis + ischemia	-	Under treatment
60	M	Severe	DM, IHD	17	Maculopapular/exanthum	Lymphocytic perivascular infiltration in dermis.	Under treatment
48	M	Critical	Nil	04	Livedo reticularis	-	Under treatment
27	M	Critical	HIV	02	Vesicular/bullous	Hyperkeratosis, necrotic epidermis, sub-epidermal blister, perivascular lymphocytic infiltration in dermis; SJS/TEN-like picture.	Death
73	M	Critical	DM, HTN, IHD	10	Maculopapular/exanthum	Chronic inflammatory lymphocytic infiltration in dermis; necrotic areas in epidermis.	Under treatment
61	M	Critical	DM, HTN, IHD, COPD	05	Livedo reticularis	Perivascular lymphocytic infiltration in dermis.	Under treatment
72	M	Severe	DM, HTN, IHD	20	Perniosis	-	Discharged
60	F	Mild	Nil	08	Ecchymosis/purpura	-	Under treatment
55	M	Critical	DM	17	Perniosis + ischemia	-	Death
72	M	Critical	DM, HTN	07	Ecchymosis + ischemia + blister	-	Death
59	M	Severe	Asthma	10	Ecchymosis/purpura	-	Discharged
80	M	Severe	DM, HTN, IHD, COPD	16	Ecchymosis/purpura	-	Death
39	M	Severe	Nil	19	Ecchymosis/purpura	-	Discharged
55	M	Severe	DM, HTN, IHD	12	Ecchymosis/purpura	-	Discharged
70	M	Severe	Nil	18	Ecchymosis/purpura	-	Discharged
59	M	Severe	Nil	18	Ecchymosis/purpura	-	Discharged
77	F	Critical	DM, HTN, IHD	5	Ecchymosis/purpura	-	Death
31	M	Mild	IHD	12	Urticarial like rash	-	Discharged
48	F	Moderate	Nil	11	Ecchymosis/purpura	-	Discharged
26	M	Moderate	Nil	3	Ecchymosis/purpura	-	Discharged
65	M	Severe	HTN	12	Ecchymosis/purpura	-	Discharged
62	F	Severe	DM, HTN, IHD, COPD	16	Ecchymosis/purpura	-	Discharged
54	M	Mild	Nil	11	Ecchymosis/purpura	-	Discharged
40	M	Mild	Lymphoma	10	Livedo reticularis	Hyperkeratosis, hypergranulosis, moderate epidermal hyperplasia with perivascular lymphocytic infiltration in dermis.	Discharged
48	M	Moderate	HCV/DCLD	08	Maculopapular/exanthum	-	Discharged
52	M	Severe	Bipolar/depression	18	Maculopapular/exanthum	Orhtokeratosis, thin epidermis, loss of rete ridges, and perivascular lymphocytic infiltration with RBC extravasation in dermis.	Discharged
43	M	Moderate	Nil	15	Ecchymosis/purpura	-	Discharged
40	M	Severe	Lymphoma	-5	Livedo reticularis	Mild perivascular lymphocytic infiltration in the upper dermis; acanthosis, hyperkeratosis.	Discharged
36	M	Severe	Nil	26	Ecchymosis/purpura	-	Discharged
65	F	Moderate	CA gallbladder	06	Maculopapular/exanthum	Hyper and orthokeratosis, increased basal and suprabasal pigmentation; perivascular lymphocytic infiltration in dermis.	Discharged
62	M	Severe	Nil	22	Vesicular/bullous	-	Discharged
59	M	Moderate	HTN	10	Ecchymosis/purpura	-	Discharged
40	F	Critical	DM	08	Perniosis + livedo; reticularis + ischemia	Occasional perivascular lymphocytic infiltration, orthokeratosis, and epidermal thinning.	Death
43	M	Moderate	Nil	10	Maculopapular/exanthum	-	Discharged
85	M	Critical	DM, IHD	06	Ecchymosis/purpura	-	Discharged
43	M	Moderate	Nil	02	Ecchymosis/purpura	-	Discharged
86	M	Critical	Perforated duodenal ulcer	12	Perniosis + ischemia	-	Death
51	M	Critical	DM, HTN, IHD	18	Livedo reticularis	Hyperkeratosis, hypergranulosis, epidermal hyperplasia; papillary dermal vessels show fibrinoid necrosis, perivascular lymphocytic infiltration (vasculitis).	Death
72	M	Critical	Nil	19	Ecchymosis + ischemia + blister	-	Death
55	M	Severe	Nil	13	Ecchymosis/purpura	-	Discharged
70	M	Moderate	Nil	05	Maculopapular/exanthum	-	Discharged
72	M	Critical	DM, HTN	14	Ischemic gangrene	Mild acanthosis increased basal pigmentation and vacuolated keratinocytes; dermal edema, congested blood vessels, extensive RBC extravasation throughout the dermis.	Death
52	F	Critical	Obesity, DM	07	Vesicular/bullous	Focal areas of epidermal necrosis attached to the underlying dermis, which, had acute on chronic inflammatory infiltration.	Death

Amongst patients with specific skin findings, ecchymosis/purpura was observed in a total of 50% (n=22), either in combination with ischemia and blister (4.5%, n=2) or in isolation in 45.4% (n=20). Of the total 22 patients with ecchymosis/purpura, 63% (n=14) received enoxaparin injections while 36% (n=8) did not. Mild or moderate thrombocytopenia (< 150×106/ml - ≥ 50 × 106/ml) was seen in 59% (n=13) of patients with ecchymosis/purpura. Thrombocytopenia, without enoaxaparin, was noted in 22.7% (n=5/22), while thrombocytopenia with enoaxaparin was seen in 31.8% (n=7/22) of ecchymosis/purpura patients. Similarly, 27.2% (n=6/22) received enoaxaparin but had no thrombocytopenia, while 13.6% (n=3/22) of ecchymosis/purpura patients had neither enoaxaparinnor thrombocytopenia. Maculopapular (exanthem) rash was seen in 18% (n=8). Livedo reticularis involving acral areas was present in 16.2% (n=7) patients, either alone (9.2%, n=4) or in combination with perniosis and ischemic changes, in 6.9% (n=3). Perniosis was found in 15.9% (n=7) in total, either alone (4.5%, n=2) or in combination with ischemia (4.5%, n=2 each) or livedo reticularis and ischemia (6.8%, n=3). Ischemic/gangrenous changes were seen in 16.2% (n=7) in total, either in combination with perniosis (11.3%, n=5), livedo reticularis (6.8%, n=3) or ecchymosis (4.5%, n=2). Vesiculo-bullous/blistering lesions were observed in isolation 4.6% (n=2) or in combination with ischemic changes in 4.6% (n=2), while 9.2% (n=4) in totality. Lastly, Urticarial lesions were seen in one patient (4.6%).

Amongst 13 patients who manifested nonspecific findings, herpes labialis constituted 23% (n=3), while bedsores, dermatitis passivata, and xerosis constituted 15.3% (n=2) of the lesions. Nasogastric tube-induced ulcerations, oral ulcerations, eczema, and vitiligo were seen in one patient each.

Our study found a significant association (p=0.03) between COVID-19 disease severity and the prevalence of specific skin lesions. Amongst patients with specific findings, only 9% (n=4) had mild cases of COVID-19. On the contrary, the disease severity was moderate in 20.4% (n=9), severe in 38.6% (n=17), and critical in 31.8% (n=14) (Table 2).

**Table 2:** Relation of specific skin manifestation with severity of COVID-19 disease. Significance level < 0.05, Cramers V value given for significant outcomes. Outcomes with adjusted residual > 1.96 are shown in bold

Disease	Presence	Grade of Illness				Total	p-value	Cramers V value
		Mild	Moderate	Severe	Critical			
Ischemia/gangrene	No	4	9	16	8	37	0.010*	0.508
	Yes	0	0	1	6	7		
Livedo Reticularis	No	3	9	16	9	37	0.062	.
	Yes	1	0	1	5	7		
Ecchymosis	No	2	4	7	9	22	0.619	.
	Yes	2	5	10	5	22		
Perniosis	No	4	9	14	10	37	0.243	.
	Yes	0	0	3	4	7		
Maculopapular rash	No	4	5	14	13	36	0.102	.
	Yes	0	4	3	1	8		
Urticaria	No	3	9	17	14	43	0.017*	0.482
	Yes	1	0	0	0	1		
Vesiculobullous	No	4	9	16	11	40	0.252	.
	Yes	0	0	1	3	4		

Ischemia/gangrene was significantly associated with disease severity (p=0.010, Cramér's V=0.550, adjusted residual=3.3) and mortality (p<0.001, Cramér's V=0.550). More than 50% (n=5) of deaths occurred in patients with such lesions (Table 3).

**Table 3:** Relation of specific skin manifestation with outcome of COVID-19 disease. Significance level < 0.05, Cramér's V given for significant outcomes

Disease	Presence	Outcome		Total	p-value	Cramér's V
		Discharge	Death			
Ischemia/gangrene	No	33	4	37	<0.001*	0.550
	Yes	2	5	7		
Livedo reticularis	No	30	7	37	0.562	.
	Yes	5	2	7		
Ecchymosis	No	17	5	22	0.709	.
	Yes	18	4	22		
Perniosis	No	31	6	37	0.109	.
	Yes	4	3	7		
Maculopapular rash	No	27	9	36	0.113	.
	Yes	8	0	8		
Urticaria	No	34	9	43	0.608	.
	Yes	1	0	1		
Vesiculobullous	No	34	6	40	0.005*	0.428
	Yes	1	3	4		

Livedo reticularis had a higher than the expected count in the critical disease category (adjusted residual=2.5), though the association was not statistically significant (p=0.062). Vesiculobullous lesions were associated with higher mortality (n=3/9, p=0.005, Cramér's V=0.428) (Table 3).

Around 50% (n=4) of maculopapular exanthem and 65% (n= 13) of ecchymosis/purpura were seen in severe or critical cases. However, the statistical analyses of both these lesions were non-significant in relation to disease severity and mortality. Urticaria was significantly associated with low disease severity (Table 2).

The mean age of patients with specific skin manifestations was 56.86 ± 15.81, while that of patients either with nonspecific/without any skin manifestations was 42.58 ± 16.96. This difference is highly significant, with a p-value < 0.001 (independent samples t-test). Analysis of age against individual specific skin manifestation shows that old age (>60 years) is significantly associated with ecchymosis (p=0.038), maculopapular exanthem (p=0.021), and vesiculobullous rash (p=0.029). Age showed no association with ischemia/necrosis, livedo reticularis, perniosis or urticaria (p=0.067, 0.992, 0.335, and 0.542, respectively) (Table 4).

**Table 4:** Age distribution of sample in relation to specific skin manifestation Significant p-values are marked in bold.

Age Group	Ischemia			Livedo reticularis		Purpura/ Ecchymosis		Perniosis		Maculo-papular rash		Urticaria		Vesiculo bullous rash	
	No		Yes	No	Yes	No	Yes	No	Yes	No	Yes	No	Yes	No	Yes
Young (N=149)		146	3	144	5	137	12	145	4	146	3	148	1	148	1
Old (N=55)		51	4	53	2	45	10	52	3	50	5	55	0	52	3
p-value		0.067		0.992		0.038		0.335		0.021		0.542		0.029	

**Figure 5:**
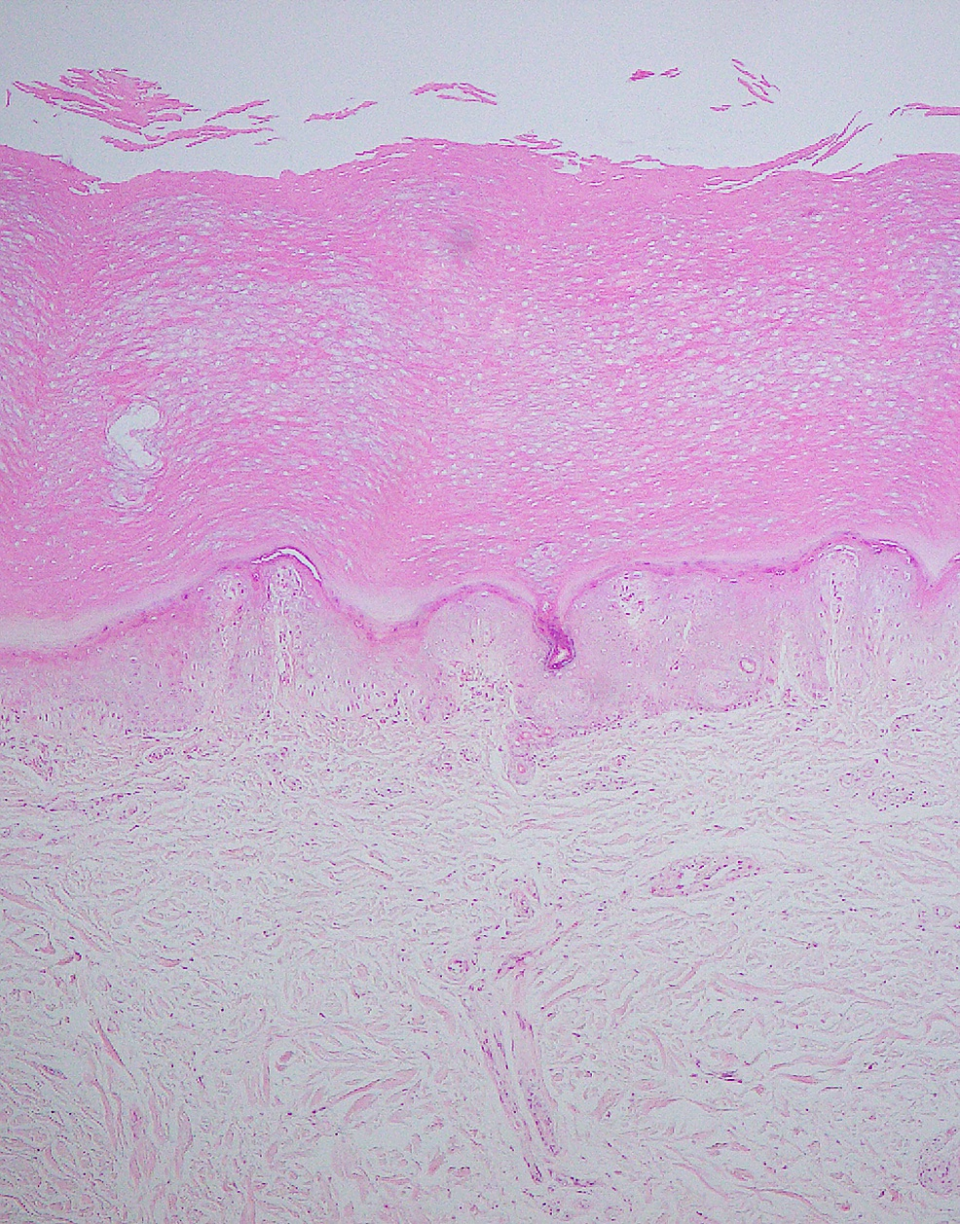
Biopsy of livedo reticularis on foot, showing some of the blood vessels with fibrinoid necrosis, congestions with extravasation of RBCs and mild perivascular lymphocytic infiltration in papillary dermis, consistent with lymphocytic vasculitis. H&E stain, 10x magnification

Histopathological examination of livedo reticularis, in one sample, showed fibrinoid necrosis, vascular congestion, extravasation of RBCs and mild perivascular lymphocytic infiltration in the papillary dermis, consistent with lymphocytic vasculitis, while the lower dermis was unremarkable (Figure 5). Five samples exhibited hyperkeratosis, hypergranulosis and perivascular lymphocytic infiltration in the upper dermis. Biopsy couldn’t be performed in one case.

**Figure 6:**
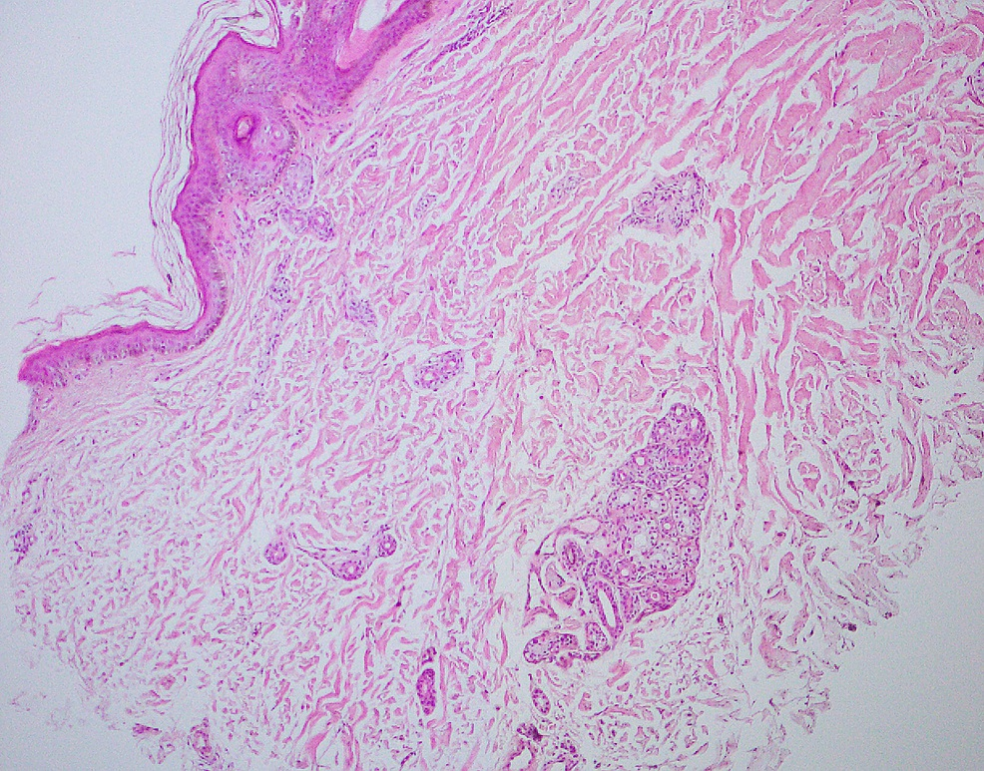
Biopsy of maculopapular rash on the forearm, showing orthokeratosis, thinning of epidermis with loss of rete ridges along with perivascular lymphocytic infiltration with RBCs extravasation in dermis. H&E stain, 10x magnification

Five samples exhibited hyperkeratosis, hypergranulosis, and perivascular lymphocytic infiltration in the upper dermis. Biopsy couldn’t be performed in one case. In the case of maculopapular rash, one biopsy manifested orthokeratosis and the dermis had perivascular lymphocytic infiltration with collagen fibers separated by mucinous material (mucinous degeneration of dermis). In another sample, orthokeratosis, thinning of the epidermis with loss of rete ridges along with perivascular lymphocytic infiltration with RBC extravasation in the dermis, was seen (Figure 6).

**Figure 7:**
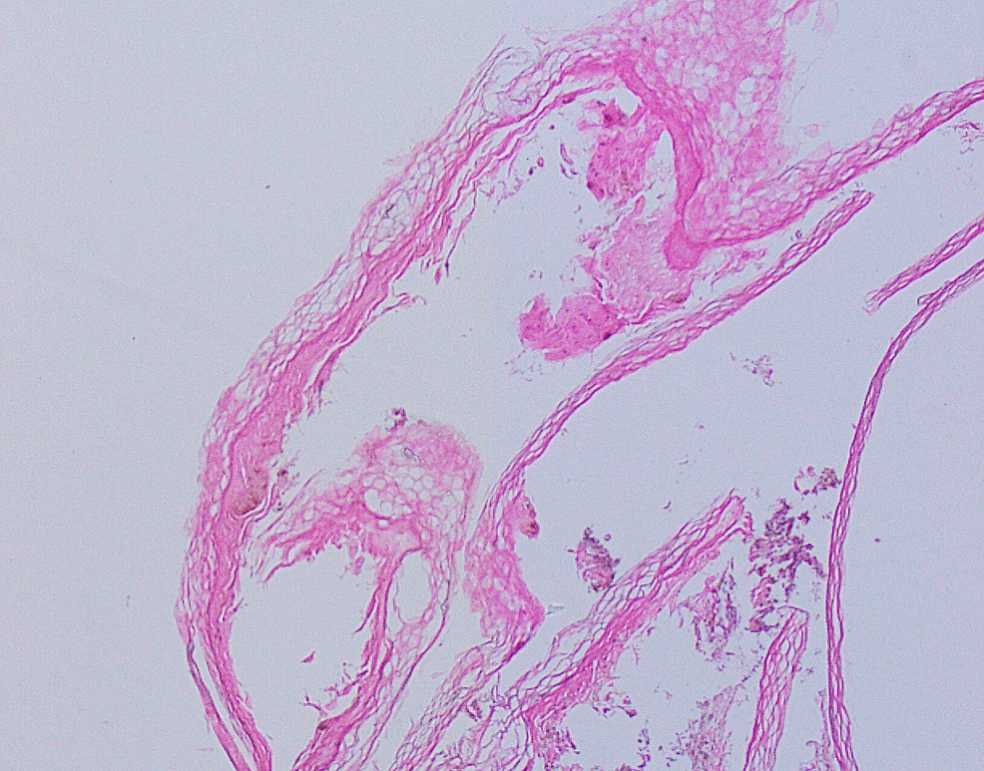
Biopsy of bullous/blister tissue from patient shown in Figure 1, showing hyperkeratosis with underlying necrotic epidermis, separated from dermis via a sub-epidermal blister. The dermis showed perivascular lymphocytic infiltration. H&E stain, 10x magnification.

Three samples showed mild perivascular lymphocytic infiltration in the dermis. Biopsies couldn’t be performed in the rest of the three cases. In a sample of necrotic plaque, the epidermis showed follicular plugging, mild acanthosis, increased basal pigmentation and vacuolated keratinocytes. There was dermal edema with congested blood vessels and extensive RBC extravasation throughout the dermis. The biopsy of bullous/blister tissue from one patient manifested hyperkeratosis with the underlying necrotic epidermis separated from dermis via a sub-epidermal blister. The dermis revealed perivascular lymphocytic infiltration (Figure 7).

**Figure 8:**
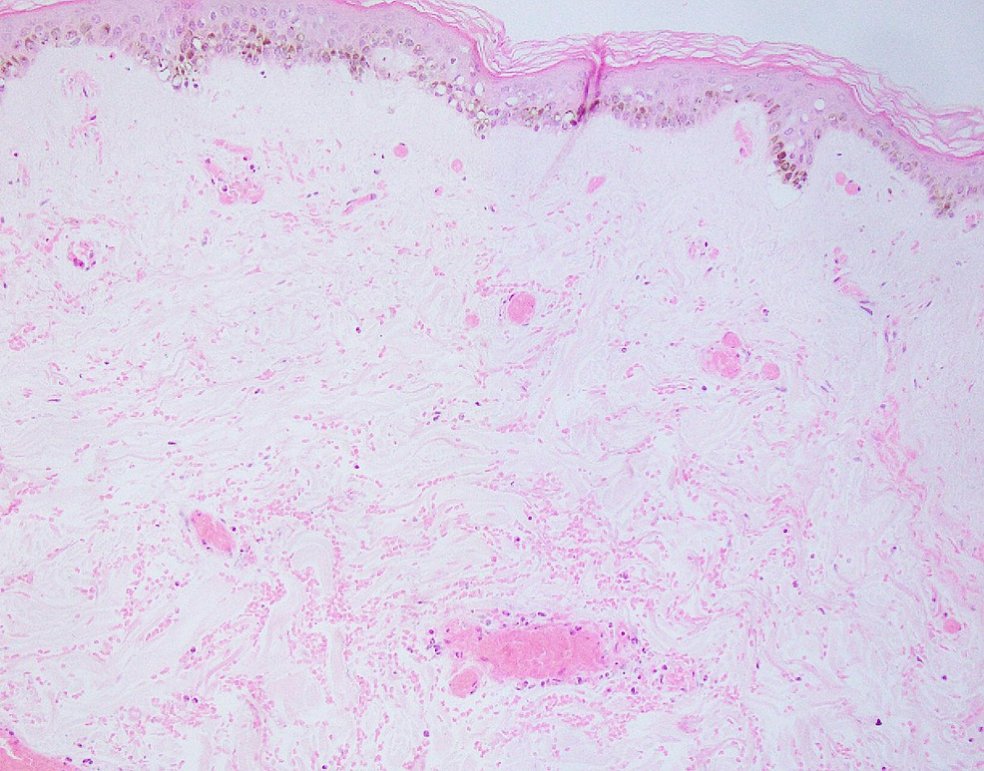
Biopsy of an ischemic plaque on forearm, epidermis show mild acanthosis, increased basal pigmentation and vacuolated keratinocytes. There is dermal edema with congested blood vessels and extensive RBCs extravasation throughout the dermis. H&E stain, 40x magnification.

Another sample of tense blisters on the face and limbs showed focal areas of epidermal necrosis attached to the underlying dermis, which in turn had acute on chronic inflammatory infiltration (Figure 8).

Histopathological examination was not done for ecchymosis/purpura, perniosis, urticaria and nonspecific skin manifestations.

## Discussion

Our study chronicles several cutaneous patterns associated with COVID-19, indicating that a considerably large group of patients may have dermatological manifestations, appearing at different instances in the course of disease at varying levels of severity, likely contributing to defining the prognosis of the disease. The description of certain histological patterns in our study may further enhance our knowledge about these manifestations. With a large number and distribution of participants, all of them testing positive for COVID-19, our sample is truly representative of the distribution of cutaneous lesions in COVID-19. Our attempt to describe the skin lesions in patients with mild to critical disease, as well as exclusion of suspected patients and testing for other respiratory viruses, reduced the chances of a bias in the results further. It is possible that some of the specific findings may have causes other than COVID-19, an example being clexane-induced ecchymosis. We tried to rule out those via carefully tracking history and examinations where an alternate cause could be suspected. Drug-induced rashes were ruled out by tracking history, careful analysis of the cutaneous findings, and performing peripheral eosinophil counts as well biopsies where feasible.

The majority of the cutaneous manifestations we observed appeared late in the course of the disease. One of the possible mechanisms behind this could be a delayed immune-mediated reaction to the virus in genetically predisposed individuals [13, 14].

Ecchymosis/purpura in the majority of patients appeared late in the disease course (second week or beyond) making it an unusable clinical feature in early diagnosis of COVID-19. We found a significant association of these lesions with a higher age group, not reported by earlier studies, to the best of our knowledge. Though statistically insignificant, two-thirds of these lesions were seen exclusively in severe/critical cases. Unlike a Turkish study [15] we noted a possible connection of ecchymosis/purpura with COVID-19 induced thrombocytopenia in 22.7% (n=5/22) patients, who had thrombocytopenia without clexane administration. Thrombocytopenia itself may be associated with a high risk of severe COVID-19 disease and mortality [16] thus serving as a clinical indicator of worsening disease during hospitalization. Other unexplained mechanisms involving vascular damage possibly via immune dysregulation, vasculitis, or thrombosis [17, 18] could be implied in 13.6% (n=3/22) of ecchymosis/purpura patients, who neither had thrombocytopenia nor clexane.

Maculopapular lesions tend to concur with other symptoms appearing in the first or the second week of illness. We found a statistically significant association of old age (>60 years) with the presence of a maculopapular rash. This was consistent with a systemic review [19] which noted the mean age of patients with maculopapular rash to be 60.4 years. In contrast to a Spanish study [20], we didn’t find these lesions to be either exclusive to severe/critical disease or associated with any mortality in our sample. Histopathological features of maculopapular lesions in our study are somewhat consistent with descriptions provided by earlier case reports of such lesions [21, 22].

Contrary to others [13], we found a higher incidence of livedo reticularis in our COVID-19 disease sample. The rash appeared in the first week of illness in most cases. Consistent with a Spanish study [20], mostly elderly patients manifested such findings in our sample. A higher than expected number of livedo reticularis patients presented with severe/critical COVID-19 disease. Although statistical significance was not found, further research is warranted to ascertain the above-mentioned relationship. Livedo reticularis may either be the primary manifestations of the disease or may point towards complications leading to vascular occlusion, as COVID-19 has already been linked to coagulation anomalies and vascular damage [23]. The presence of fibrinoid necrosis in papillary dermal vessels with congestion, RBC extravasation, and mild perivascular lymphocytic infiltration in a biopsy of livedo reticularis in our study, further augments this treatise. Livedo reticularis also exhibited hyperkeratosis, epidermal hyperplasia, hypergranulosis, and perivascular lymphocytic infiltration with RBC extravasation in the upper dermis in five biopsy specimens. A systemic study that compiled a histological description of livedo reticularis rash by Li et al. [13] reported similar histological findings. Livedo reticularis may help raise suspicion of COVID-19 as it appears earlier in the disease course, thus helpful in its diagnosis, albeit in a minority.

Pseudo-chilblain appears like perniosis. Considering that they customarily appear in July’s warm weather, with the average high temperature being 35.7⁰C [24], a noteworthy increased incidence as perceived by dermatologists, all patients being COVID-19 positive and with no previous history of such lesions, a COVID-19 link becomes highly plausible. The findings are consistent with other studies that reported perniosis-like lesions in 318 cases from eight countries [25], negating some earlier assumptions that considered these lesions to be induced by cold temperature and immobility [8]. Pseudo-chilblains appeared later, either alone or in combination with ischemic/blistering lesions in our sample. Consistent with a Spanish study [20], their association with disease severity was statistically insignificant. 

Ischemic/gangrenous lesions appeared late in the disease course, involving fingers, digits, and even whole limbs (n=2) or forearm skin (n=2) in some cases. Unlike previous studies [20], we think that these dry gangrenes could appear at any age and are particularly high in severe/critical cases. Our research correlates these findings with a bad prognosis and high mortality as reported by other limited-scale observations [26]. The presence of ischemia/gangrene in COVID-19 disease considerably augments the concept of a synergy between vasculitis and hypercoagulable states induced by COVID-19, as reported in earlier studies [9, 27].

Vesiculo bullous lesions might appear early or later in the disease course. They were significantly associated with higher mortality (n=3/9) in our cohort. We found no studies that correlated these lesions to COVID-19 mortality. Though statistically insignificant, we observed these to be far more frequent in severe/critically ill patients. Their incidence was far less compared to other skin manifestations in our study.

Consistent with a previous study [20], we think that urticarial lesions may be accredited to many causes. They do not predate other symptoms and hence, are unhelpful in making a diagnosis.

Apart from offering specific systemic therapy to treat COVID-19, clinicians and nursing staff should also give attention to maintaining general skin health and hygiene, to prevent and treat non-COVID-19 related skin issues such as dermatitis passivata, bedsores, xerosis, oral ulcers, and nasogastric tube induced ulcers [6, 7]. As observed in our study, a lot of patients, due to a lack of knowledge regarding this novel disease, didn’t know whether to follow the usual skincare routine and take showers or not. COVID-19 patients are especially prone to have neglected skin hygiene due to prolonged and isolated in-hospital stays where patient and care provider interaction is minimal due to the highly infectious nature of the virus.

COVID-19 appears to be an unusual viral infection, as it can exhibit several clinical patterns [5] of dermatological manifestations. Unlike a previous study [20], a lot of our observed skin patterns did coexist in many of our patients.

As a whole, it could be stated that dermatological manifestations have a tendency to frequent more in patients with a severe/explosive disease course. The minimal presence of skin manifestation in mild/moderate COVID-19 cases that we report is consistent with a study in patients residing in the Tibetan plateau, where the disease course was generally mild in almost all patients [28].

The uniform and direct involvement of dermatologists/residents in the triage, admission, care as well as the search for skin manifestations at all times and levels in our study, may have translated into an accurate reporting of the incidence of these skin findings and their association with disease severity. Especially when a few previous attempts have either reported a highly skewed incidence of skin findings involving almost all the patients [29] or reported minimal involvement of skin where observation was done by non-dermatological staff [30], others simply lacked photographic and/or histologic data [3].

The description of clinicopathologic features augments our existing knowledge on some of the patterns and provides an insight into the histology of cutaneous findings associated with COVID-19. Further structured clinical, molecular, serological, and histopathological studies are required to ascertain and explain the dermatological manifestations and their possible utilization for better management of the patients with COVID-19.

## Conclusions

The majority of dermatological manifestations of COVID-19 such as perniosis, ischemia, livedo reticularis, and ecchymosis/purpura are more frequent in severe/explosive cases than other manifestations such as vesiculobullous, ecchymosis, and maculopapular exanthem in the older age group. Ischemic/gangrenous and vesiculobullous lesions herald a bad prognosis with high mortality. The presence of these dermatological manifestations could help set prognostic criteria in the future.

There is a possible connection of ecchymosis/purpura with COVID-19-induced thrombocytopenia. Histopathological features vary according to the skin lesions. They may be specific or nonspecific. Attention should be given to maintaining general skin health and hygiene in the prevention and treatment of non-COVID-19 related skin issues.
